# Beetle pollination and flowering rhythm of *Annona coriacea* Mart. (Annonaceae) in Brazilian cerrado: Behavioral features of its principal pollinators

**DOI:** 10.1371/journal.pone.0171092

**Published:** 2017-02-02

**Authors:** Marilza Silva Costa, Ricardo José Silva, Hipólito Ferreira Paulino-Neto, Mônica Josene Barbosa Pereira

**Affiliations:** 1 Departamento de Entomologia, Universidade Federal de Viçosa (UFV), Viçosa, MG, Brazil; 2 Laboratório de Zoologia, Centro de Pesquisa, Estudos e Desenvolvimento Agroambiental (CPEDA), Universidade do Estado de Mato Grosso (UNEMAT), Tangará da Serra, MT, Brazil; 3 Programa de Pós-Graduação em Entomologia, Departamento de Biologia, Faculdade de Filosofia, Ciências e Letras de Ribeirão Preto, Universidade de São Paulo, Ribeirão Preto, SP, Brazil; 4 Curso de Ciências Biológicas e Laboratório de Ecologia da Polinização, Evolução e Conservação (LEPEC) da Universidade do Estado de Minas Gerais (UEMG), Unidade Passos, MG, Brazil; 5 Departamento de Agronomia, Laboratório de Entomologia, Centro de Pesquisa, Estudos e Desenvolvimento Agroambiental (CPEDA), Universidade do Estado de Mato Grosso (UNEMAT), Tangará da Serra, MT, Brazil; Universidade de Sao Paulo Faculdade de Filosofia Ciencias e Letras de Ribeirao Preto, BRAZIL

## Abstract

The conservation and sustainable management of *Annona coriacea* requires knowledge of its floral and reproductive biology, and of its main pollinators and their life cycles. In this work, we analyzed these aspects in detail. Floral biology was assessed by observing flowers from the beginning of anthesis to senescence. The visiting hours and behavior of floral visitors in the floral chamber were recorded, as were the sites of oviposition. Excavations were undertaken around specimens of *A*. *coriacea* to determine the location of immature pollinators. Anthesis was nocturnal, starting at sunset, and lasted for 52–56 h. The flowers were bisexual, protogynous and emitted a strong scent similar to the plant´s own ripe fruit. There was pronounced synchrony among all floral events (the period and duration of stigmatic receptivity, release of odor, pollen release and drooping flowers) in different individuals, but no synchrony in the same individuals. All of the flowers monitored were visited by beetle species of the genera *Cyclocephala* and *Arriguttia*. Beetles arrived at the flowers with their bodies covered in pollen and these pollen grains were transferred to the stigmata while foraging on nutritious tissues at the base of the petals. With dehiscence of the stamens and retention within the floral chamber, the bodies of the floral visitors were again covered with pollen which they carried to newly opened flowers, thus promoting the cycle of pollination. After leaving the flowers, female beetles often excavated holes in the soil to lay eggs. Larvae were found between the leaf litter and the first layer of soil under specimens of *A*. *coriacea*. *Cyclocephala* beetles were the main pollinators of *A*. *coriacea*, but *Arriguttia brevissima* was also considered a pollinator and is the first species of this genus to be observed in Annonaceae flowers. *Annona coriacea* was found to be self-compatible with a low reproductive efficiency in the area studied. The results of this investigation provide ecological data that should contribute to the conservation and economic exploitation of *A*. *coriacea*.

## Introduction

The pollination ecology allows the understanding of the structure of natural plant communities [1–4] and provides information about the shape of the flowers, which makes it possible to characterize the mechanisms of pollination and adaptation of visitors to the flower [5]. Moreover, the relationship between plant and pollinator are also important in the structuring of communities, and can influence the spatial distribution of plants in the richness and abundance of species [3, 4, 6, 7]. In the Cerrado, known for its great wealth of plant species [3, 8–12] are studies on the reproductive systems, sexual and pollination, which, these are fundamental to the understanding of the biological processes [3, 13–15].

The Annonaceae family is ninth in the representation of the Brazilian Cerrado [11, 16], and has some peculiarities about the pollination process, in which the beetles are the main agents [2, 17–19]. One of reproductive strategies to attract pollinators in Annonaceae is the release of odors that are produced by osmophores in the internal base of the petals. During the night, there is flowering and temperature rise in anthesis, accompanied by the production of odor [2, 19–27] with consequent volatilization and attraction of pollinating beetles. These penetrate the flower forcing entry through the petals, and remain inside for a period ranging from hours to a few days, feeding on pollen and/or nutritional tissues of the petals, at the same time, using the flower as a place of shelter and a copula. This protection of beetles inside the flower is due to the presence of a floral or pollination chamber formed by the inner petals that curve towards the flower’s center during anthesis [3, 19, 21, 22, 28–31]. This whole set of strategies, including morphological and physiological adaptations that *Annona* flowers have for visitors, gives them greater chances of pollination success [2, 20, 21, 23].

Many *Annona* species have considerable economic importance, essentially *A*. *muricata* L. (soursop), *A*. *squamosa* L. (sugar apple), *A*. *cherimoia* Mill. (cherimoya), *A*. *crassiflora* Mart. (marolo) are annonaceous species known to produce very tasty fruits appreciated by the rural and urban populations. Their pulps can be consumed raw as ingredients to prepare ice creams, juices, jams, jellies, liquors, fillings, cake and other traditional culinary recipes from the region. In addition, the corresponding trees have potential as urban trees. *Annona*. *coriacea* Mart. (araticum-liso) has very close economic potential of these species previously described, though still largely unexplored. There is even a lack of information regarding the floral biology and what its main pollinators [26, 31].

*Annona coriacea*, which is widely distributed in the Brazilian Cerrado [32], has fleshy fruits consisting of important food resource for native fauna [33], and highly appreciated by the local population. It is a species considered as primary in the ecological succession process, and its occurrence in these areas attracts pollinators and seed dispersers, and is an important component in environmental conservation and the restoration projects. However, Cerrado in Mato Grosso State is losing ground to agriculture, so that natural areas are increasingly fragmented, disconnected and degraded. In addition, the use of insecticides on crops has directly affected the populations of pollinating insects of many species of native plants [34, 35], compromising their reproductive success and, hence, upsetting the natural occurrence not only of *A*. *coriacea* [35], but also other plant species.

Therefore, the knowledge of floral and reproductive biology, floral morphology, pollination mechanisms and main pollinators of *A*. *coriacea* and their life cycles are extremely important for the conservation and sustainable management of these species. Thus, this work aimed to: (1) verify the anthesis period of araticum in Cerrado of Mato Grosso; (2) identify the main pollinators; (3) record the behavior of pollinators during flowering and (4) determine the reproductive system.

## Material and methods

### Study area

The study was conducted from November 2005 to April 2006. We observed 35 plants of *A*. *coriacea* of reproductive age located mainly in the Cerrado area, from Fazenda Três Rios to Nova Marilândia (14°23'S; 57°42'W), state of Mato Grosso, Central West region of Brazil. The climate is tropical, with average temperature of 25.6°C, average annual rainfall of 1895 mm and altitude of 467m [36]. The study area is located inside Mr. Leandro Salles Nogueira property, who gently gave us permission to conduct the study there. Our studies did not involve endangered or protected species.

### Floral biology

Floral events had occurred prior to initiation of the study, and were determined by direct observations in 11 floral buds distributed in four plants. The floral buds that were followed from flower opening up to the dehiscence of the period of the petals and flowers were observed in 6-hour intervals until the moment of dehiscence. The flower characteristics were recorded: color, length, width and thickness of the outer and inner petals; color and dehiscence of the stamens; color, diameter and viscosity of the stigmata; presence or absence of odor, and distance between the petals. The measurements were performed with the aid of a caliper and a pachymeter.

### Pollinator behavior

During seven consecutive days (15–21 January 2006), 28 flowers distributed in 13 plants of *A*. *coriacea* were observed during the period from 17:00 to 06:00 hours, during which the flowers release their odor in order to attract the beetles. As the flowers did not come into anthesis when it would have been possible to monitor them individually for 10 minutes every 6 hours, the total time for all the flowers that were in anthesis each night, were observed. After all the flowers had been monitored, a new cycle of observation was initiated by repeating several cycles overnight. Finally, it resulted to a total of 91 hours of observation.

The methodology used to describe the beetle's behavior was adapted from Altmann [37] which was to write down all instances of certain classes of behavior in all members of the group during each period of observation. The observed behavior among these classes included the arrival at and exit from the flower, drive in bloom, removal of stamens, immobility, food, and arrival and departure with pollen on the body. All these behaviors were previously established, avoiding any interference with the behavior of floral visitors, using flashlights covered by red cellophane minimizing the influence of light on the behavior of the beetle within the floral chamber. Beetles were photographed and collected for identification after the end of the observation period.

### General aspects of pollinator behavior after visits to flowers

The fate of 16 beetles after mating inside the flower was observed in order to register the oviposition sites of females. After this finding and still within the *A*. *coriacea* flowering period in the area, eight excavations of 1×1×1 meters in the ground about 5 meters away from the plant, and also within 100 meters of these plants were carried out. All Coleoptera larvae that were found were collected, photographed and identified based on the distribution by the raster, which corresponds to the tenth segment of their abdomen.

### Reproductive system

Experiments to determine the reproductive system were conducted from December 2005 to February 2006. Flower buds (n = 100) in the pre-anthesis phase distributed in 17 plants were bagged in fine mesh nylon fabric (< 1 mm) to prevent the visit of pollinators, were submitted to the following pollination treatments: (1) spontaneous self-pollination (flowers were simply bagged to check the occurrence of automatic self-pollination (n = 67); (2) Cross-pollination of emasculated flowers (all stamens of flowers were previously removed to avoid self-pollination and were pollinated with pollen derived from flowers of other plants (n = 5); and (3) control (to evaluate the efficiency of natural pollination), some flowers were only marked and not bagged to allow visitation by natural pollinators (n = 28).

The production of fruit for each treatment was monitored weekly until 3 months after its complete formation. It also calculated the reproductive efficiency index (REI) [38], through the relationship between the percentage of fruiting by open pollination (control) and manually cross-pollinated flowers. According to Ruiz and Arroyo [38], reproductive efficiency is estimated by the relative effectiveness of natural pollination. Finally, we calculated the Index of Self-Incompatibility (ISI: relation between the percentage of fruits produced through self-pollinations/percentage of fruits produced through cross-pollinations). Below 0.25, the plants are considered self-incompatible [39].

## Results

**Fig 1:**
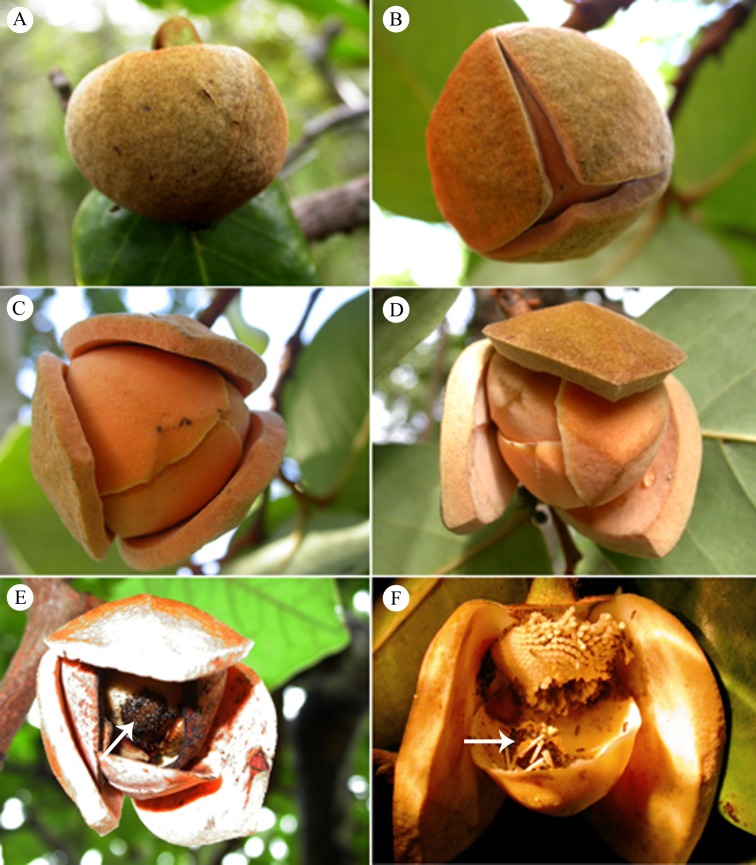
Floral event *A*. *coriacea*. (A) Floral button in pre-anthesis; (B) Flower opening in process, and expansion of petals 12 hours after the onset of anthesis; (C) Flower fully open 24 hours after onset of anthesis; (D) Flower in female stage 48 hours after anthesis; (E) Flower also in female phase, also 48 hours after anthesis, but petals spaced manually to allow the inside of the floral chamber to be displayed, stamens assembly further strongly adhered to each other without releasing, and yet with pollen-receptive stigmas. Note the production of exudate, a bright and viscous substance on the stigma that has function of adhering to the pollen grains (arrow); (F) Flower male phase 52 hours after anthesis, with stamens falling off and starting release of pollen, and droop of stigmatic (arrow) marking the end of the female phase (outer and inner petals partially removed for better viewing).

The commencement of anthesis in *A*. *coriacea* was characterized by a slight separation between the apices of the outer petals, when it is possible to see part of the inner petals. These flowers do not expose the gynoecium and androecium even when open, since the petals recurve towards the flower center, forming a floral chamber. Anthesis occurred during the night (from 17:00 hours), and the duration of flowers ranged 52–56 hours since the separation of the outer petals from the total detachment of the stamens of the receptacle (Fig 1).

At the beginning of the observations, the flower buds were in advanced stages of development (pre-anthesis), and kept the outer petals joined to each other (Fig 1A). The onset of anthesis was marked by a slight separation of the outer petals on average not more than 5.5 ± 2.54 mm (Fig 1B). At this stage, the outer petals measured 23.9 ± 3.72 mm long by 28.2 ± 3.60 mm wide, and had a greenish orange color. Twenty four hours after the onset of anthesis, the outer petals were completely expanded, more spaced out and more flexible, exposing the inner petals overlapping and less thick than the external petals. At this stage, the outer and inner petals had an orange staining (Fig 1C). Between 24 and 48 hours, it could be seen that the flower was functionally female (Fig 1D), and exuded a soft, sweet odor, and had receptive stigmas releasing a viscous, bright exudate (Fig 1E).

After 48 hours, there was a slow swelling of sterile cells that surrounded the stigmas, and about two hours later came the fall of the stigmatic head, characterizing the end of the female phase. In addition, there was loosening of the many stamens that make up the androecium, and a subsequent release of pollen, thus starting the male phase (Fig 1F). There is a gap between the female and male phases, and therefore, there is no overlap between them. The time required from the start of the dehiscence of stamens until the start of the pollen being released is about 2 hours. Already the male phase, understood to be from the release of pollen until the droop of the flower containing pollen trapped in its floral camera and floral visitors lasted about 8 hours.

Plants of *A*. *coriacea* that have more than one flower in anthesis presented a synchrony between all floral events: stigmatic receptivity hours, release of odor, loosening and detachment of stamens, pollen release and drooping flowers. However, this timing was not observed among individuals in the population, but only intraindividual.

It was often seen that flowers started the anthesis process at different times of the day, but all the flowers were actually functional, exuding strong odor, very exudate on stigmata and extended petals, and allowing entry of beetles in the late afternoon and early evening, which is the period that flower visitors came to the flowers. Generally, the flowers were functionally female in the period from 17:00 to 22:00 hours, and the onset of male function with pollen release occurred between 23:00 and 2:00 hours.

Beetles visited all the 28 flowers observed. The total number was 39 beetles, exclusively scarabs, and each species was represented by 13 individuals of *Cyclocephala atricapilla* Mannerheim (1829), 12 *Cyclocephala undata* Olivier (1789), 4 *Cyclocephala octopunctata* Burmeister (1847), 4 *Cyclocephala ohausiana* Hoehne (1923) and 6 *Arrigutia brevissima* Arrow (1911). The number of visitors per flower varied from one to five beetles.

**Fig 2:**
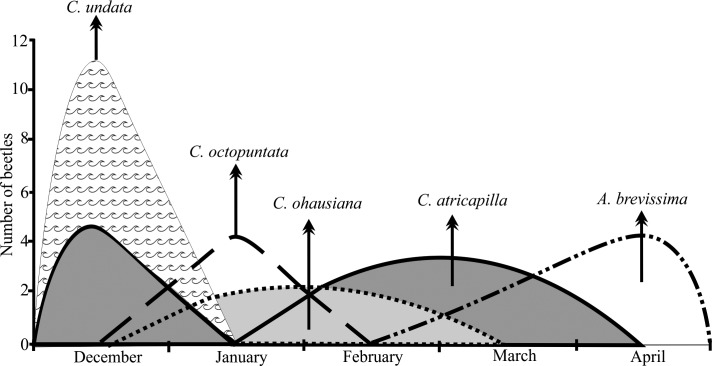
Abundance (n) and occurrence period (months) of pollinators during flowering *Annona coriacea* (Annonaceae) in the Cerrado of Mato Grosso in December 2005 to April 2006.

All species of beetles made legitimate visits to the flowers of *A*. *coriacea*, that is, they entered with pollen stuck to the body and left the flowers with their bodies covered by pollen. While remaining within the floral chamber, the visitors touch the pistil and pollen gets deposited on the stigma, which behavior is not observable, as it would be hurtful to the reproductive organs, especially the pistil. Such visits occurred at different periods (days), differing with varying intervals, sometimes reaching up to 30 days. The various species of *Cyclocephala* showed visitor behavior to the flowers of temporally sequential *A*. *coriacea*. Although some species co-occur with one to three other species, there has always been the predominance of a particular species over the flowering period. In December, there was a predominance of *C*. *undata*, but co-occurring with *C*. *atricapilla*, which also showed a significant abundance. During the month of January, *C*. *octopunctata*, in February and March *C*. *atricapilla*. *Cyclocephala ohausiana* already occurred between the months of December and March, and was the second most abundant species. By April, there was only *A*. *brevissima*. In addition, *C*. *atricapilla* occurred in all months observed, especially in February and March. Finally, *A*. *brevissima* was occurring between February and April, but dominant in late March and throughout the month of April (Fig 2).

**Fig 3:**
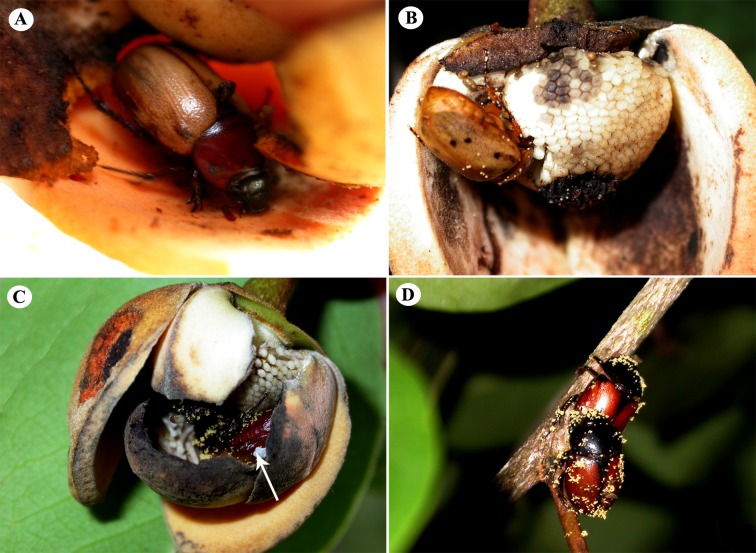
Pollinators of *Annona coriacea*. (A) *Cyclocephala atricapilla* feeding on nutritious tissues on the outside petal. (B) Flower with the external petal removed for better viewing of the floral chamber; note *C*. *ohausiana* feeding on the stamens. (C) Flower with removed outer petal; arrow indicates *C*. *atricapilla* during anther dehiscence receiving pollen that is attached on its body. (D) *Arriguttia brevissima* with the body covered with pollen.

The beetles began to get the flowers from 19:00 hours, but the higher incidence of visits (62.50%, n = 10) occurred during the period of 22:00 and 3:00 hours, always after intensification of the odor emitted by the flower. Beetles arrived at the flowers in a zigzag type of flight, forcing open the outer and inner petals to penetrate inside the floral chamber, where they remained until the drooping of the flower. Once inside the floral chamber, they fed the nutritional tissues located at the base of the inner petal and deposited pollen (Fig 3A). This action was observed throughout the night, but there was a higher occurrence of this behavior (57.14%, n = 8) also between 22:00 and 03:00 hours, when occasionally beetles were observed feeding on stamens still stuck firmly in the receptacle (Fig 3B) before they released pollen.

Most beetles arrived at the newly opened flowers with pollen covering the body and visited during the female phase, 72% (n = 36) of visits occurred between 22:00 and 3:00 hours. While remaining within the chamber floral, beetles moved around actively during this period of 18:00 and 03:00 hours. Some beetles remained inside the floral chamber, commonly in copulation until dehiscence of the petals, and left the flowers with body covered and filled with pollen grains (Fig 3C). The fall of flowers occurred at a very specific time between 18:00 and 22:00 hours corresponding to the third day since the start of anthesis, ending the phase of the functionally male flower and its dehiscence. After the senescence of flowers, beetles flew to other newly opened and functionally female flowers (receptive) with the body covered with pollen (Fig 3D), ushering in a new pollination cycle.

**Fig 4:**
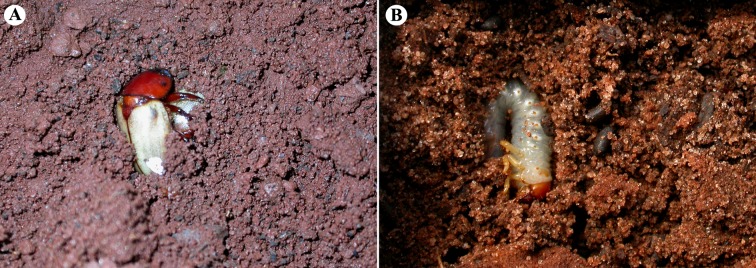
Adults and larvae *Cyclocephala*, pollinators of *Annona coriacea*. (A) Adult of *C*. *atricapilla* (Scarabaeidae: Dynastinae), digging in the ground near *A*. *coriacea* plant after the fall of araticum flower. (B) Larvae Cyclocephala found between litters and soil just below *A*. *coriacea* individuals.

After falling into the soil together with the petals, beetles (n = 11) excavated and penetrated the soil (Fig 4A). Twenty five larvae of *Cyclocephala* were detected between the litter and the first layers of the soil, feeding on decaying material (Fig 4B). Larvae were found just below the araticum cup and/or under trees next to it, a distance not exceeding 100 meters.

*Annona coriacea* presented a fruit formation rate of only 1.5% for the treatment of spontaneous self-pollination and cross-pollination to 100%. Already the flowers under natural pollination showed a fruiting rate of 42.8%. The reproductive efficiency was 0.42 (Table 1).

**Table 1:** Fruit set from controlled pollination of flowers of *Annona coriacea* Mart. (Annonaceae), in Cerrado of Mato Grosso State in December 2005 until April 2006.

Pollination Treatments	Flowers (n)	Fruits (n)	Success (%)
Spontaneous self-pollination	67	1	1.5
Cross Pollination	5	5	100
Natural pollination (Control)	28	12	42.8
Reproductive Efficacy			0.42

Reproductive efficiency—(percentage of fruits from natural pollination/percentage fruit derived from cross-pollination, *sensu* Ruiz & Arroyo 1978).

## Discussion

The *A*. *coriacea* flowers have a night anthesis lasting 52–56 hours. In *A*. *coriacea*, as in most species of Annonaceae, anthesis occurs in two distinct phases due to the time difference between the received stigmas and pollen release, thereby preventing self-pollination [22]. Most species of Annonaceae studied so far are self-pollinated, but cross-pollination is essential to greater reproductive success and increased gene flow once the flowers are dichogamy [11, 18, 30, 31].

The floral events observed in *A*. *coriacea* comprises from separation of the first whorl until the dehiscence of flowers, especially the odor release and the production of viscous substance (exudate) on the stigma, to promote the adhesion of pollen on the stigmatic surface, thus promoting pollination. These events have been studied for other Annonaceae, such as *Unonopsis guatterioides* [40], *A*. *squamosa* L.[41], *A*. *crassiflora* [28], *A*. *sericea* [20], *Xylopia aromatica* [22]and *Cardiopetalum calophyllum* [42].

Floral smells given off by *A*. *coriacea* and other species of Annonaceae act as chemical signals to attract insects to feeding sites and for breeding [43, 44]. These signs with floral morphology and behavior of visitors favor pollen transfer [30], once the release of pollen in *A*. *coriacea*, the petals fall and the beetles fly to a new flower in search of shelter, food, and, of course, for sex partners. At the moment that the senescent flowers fall to the ground capped with pollen beetles, newly opened and receptive flowers commence emitting intense odors which attract these beetles to penetrate the floral chambers, thus transferring pollen from other flowers to their stigma, promoting gene flow between flowers of different individuals both (cross-pollination) and among those belonging to the same individual (geitonogamy) [11, 19, 29, 31, 45, 46]. The pollinator is attracted to the flowers of *A*. *coriacea* and pollinate them, and they offer very nutritious tissues called "power bodies" that have cells rich in starch, lipids, tannins and mucilage [11, 46]. The power bodies are located in the basal portion of the petals [20, 24, 46].

The beetles, *C*. *atricapilla*, *C*. *undata*, *C*. *ohausiana*, *C*. *octopunctata* and *A*. *brevissima* (Scarabaeidae) consist of the fauna of pollinators of *A*. *coriacea* in Cerrado of Mato Grosso State. Due to its abundance in relation to other species, *C*. *atricapilla* was considered the main pollinator of *A*. *coriacea*. This species was observed mostly during the araticum flowering period, which may be pointed out, as suggested by Gottsberger [28] and Paulino-Neto [19, 31, 47], most common and abundant species of *A*. *coriacea* flowers, and thus, its effective pollinator, while the other species (*C*. *undata*, *C*. *ohausiana*, *C*. *octopunctata* and *A*. *brevissima*) are secondary pollinators.

With the exception of *C*. *atricapilla*, the species observed in this study, had not been reported as visitors to *A*. *coriacea*, but have been identified as pollinators of other species of Annonaceae and other families. *C*. *octopunctata* was described as the main pollinator of *A*. *crassiflora* (Annonaceae) [48], *C*. *undata* of *Duguetia ulei* (Annonaceae) [23], and *C*. *ohausiana* of *Caladium striatipes* (Araceae) [49], and *A*. *brevissima* was described as guest species of Araceae [50], which is the first to record Annonaceae.

All species observed, regardless of the time of year, exhibit similar behavioral features on the flowers in search of food, shelter and mating site [11, 19, 47, 51], but differ in the period of occurrence throughout the flowering period.

Probably the sequential occurrence of these pollinators of *A*. *coriacea* during the flowering period directly reflects upon the period of emergence of adults. Many species of Dynastinae synchronize their reproduction with the season of the cultures from which they feed [52]. During this period, the adults emerge from the ground to look for a partner for mating, and after copulation, females return to land, likely, to lay their eggs [53, 54]. The period of occurrence of each species of flower visitors was approximately 30 to 60 days, which period coincides with longevity described for *C*. *melanocephala* adults under laboratory conditions [55].

After copulating inside the floral chamber of *A*. *coriacea* and leaving the flower, the female beetles excavate areas and, likely lay their eggs in places just below the araticum tree, or near these, where there is plenty of litter, to ensure the success of the offspring, since, according to Richter [56], the larvae of Dynastinae normally feed on decaying organic matter.

In most species of Dynastinae, the larvae can be found in the soil between the months of November and March [52], and remain active for about 6 months feeding on roots of plants and organic matter [54]. It is believed that these pollinator beetles present annual life cycles, as described for *C*. *verticali* under laboratory conditions [54].

The Index of Self-Incompatibility (ISI = % of fruits produced through self pollinations / % of fruits produced through cross-pollination) was not calculated due to the lack of the artificial self-pollination treatment. According to Bullock [39], plant species with ISI ≥0.25 are self-compatible. However, there was fruit production in this present study from the treatment of spontaneous self-pollination, indicating that *A*. *coriacea* is a self-compatible species. Despite having produced just one fruit from 67 sampled flowers this species is considered self-compatible. In addition, Paulino-Neto [31] performed a thorough studied on the breeding system of *A*. *coriacea* and verified that is a self-compatible species with ISI of 0.75, inclusive considered high. Thus, Paulino-Neto [31] data agrees with our indication that *A*. *coriacea* consist in a self-compatible species.

Although the Reproductive Efficacy Index (REI) of 0.42 exhibited by *A*. *coriacea* may be considered high when compared with other tropical plant species [38, 57, 58], here it was considered low, since it presented a fruit set from natural pollination that was less than one half of that obtained by artificial cross-pollination. Many studies with native plant species found reproductive efficacy higher than 0.50 [59–62], and other studies with extremely high reproductive efficacy of 0.94 [63], and including the own *A*. *coriacea* with 1.17 with natural pollination more effective than artificial cross-pollination [31]. Thus, an REI just 0.42 may indicate a pollen limitation [62, 64–66]. This pollen limitation can be as the resultant of the high amount of self-pollination and mainly geitonogamy events under natural conditions with these beetle pollinators [38]. *Cyclocephala* pollinators may visit several flowers belonging to the same plant before flying to a new plant, thus promoting self-pollination (geitonogamy). As geitonogamy presents a fruit-set considerable smaller than cross-pollination, this can result in low reproductive success as observed. In this context, *A*. *coriacea* plants were geographically distributed in patches in the study area, which greatly favors the occurrence of geitonogamy, and probably, this is the main reason for the low reproductive efficacy registered here. Thus, in this context, it is probable that the pollen limitation observed here has a “quality” component, instead of a "quantity" component [67–69], since pollen loads delivered on stigmas have geitonogamous origin, and thus, less genetic variability, and commonly produce a lower fruit set than cross-pollinated flower [67, 70]. In addition, as all artificially cross-pollinated flowers produced fruits, the possibility of water or nutrient limitation can be playing a more important role in limiting the tree fertility was not considered.

However, the REI obtained in this study must be viewed with reservation as they were conducted few cross-pollination treatments (n = 5) compared to the control treatment (n = 24), and data may not represent the reality of this pollination system. Considering that many studies showed that *Cyclocephala* beetles are efficient long-distance pollinators, capable of transporting pollen among individuals located in isolated fragments of Cerrado [2, 18], it is possible that the REI is higher than that found here, and future studies should be conducted to confirm the reproductive effectiveness in this pollination system.

## Conclusions

Anthesis in *Annona coriacea* lasted 52–56 hours, starting synchronously at nighttime. It is a cantharophilic species, considering the flower structure and the abundance and behavior of visiting beetles. The main pollinator is *C*. *atricapilla*, and its secondary pollinators are *C*. *undata*, *C*. *ohausiana*, and C. *octopunctata* and *A*. *brevissima*. However, future studies are needed to assess the relative importance of these species of beetles as effective pollinators. By visiting the flowers, beetles feed on pollen and nutritious tissues located at the base of the petals, copulate and pollinate the flowers. After leaving the flowers, the females dig the soil and lay eggs (in the soil below araticum tree, or a distance up to 100 meters away), in places rich in leaf litter. There the larvae remain feeding on roots and decaying material until the emergence of the adult, a period coinciding with the beginning of the next flowering. *A*. *coriacea* is a self-compatible species, and the area had low reproductive efficiency (0.42) producing less fruit coming from natural pollination than those produced under treatment of cross-pollination.

This statement corroborated by a recent study of the same species of plant found reproductive efficiency of 1:17 with natural pollination more effective than artificial cross-pollinations. This low reproductive efficiency may be a result of the occurrence of large numbers of geitonogamy selfing that favored the distribution of individuals in patches in the study area, and how the plant is self-incompatible, does not produce fruit when self-pollinated. Data also indicate the occurrence of a resulting limitation of high self-pollen deposition. In this case, the pollen limitation probably has a "qualitative" rather than "quantitative" component, since data indicate that there are effective pollinators in the study area, but transferring pollen of geitonogamy origin, thus not resulting in fruits. Future studies are needed to investigate the distribution of *A*. *coriacea* in patches, the main factor responsible for this low reproductive efficiency and pollen limitation, or if there are other factors not addressed here, as the resources in the soil, such as water and/or nutrients, and climate changes that modify the mean temperatures.

Better comprehension on floral biology, flowering rhythm and floral traits are the key in achieving the effective and required conservation and management of this annonaceous species, as well as its pollinator fauna. This ecological information are crucial to greater reproductive success and increased gene flow once the flowers are dichogamous. In addition, beetle pollinators of Annonaceae species usually are very specialized, pollinating just one or few species, and they are attracted mainly by floral biology and floral features. How very often is registered sequential flowering rhythm over the months, situation in which the beetles find feeding and mating places over the year, consider the floral rhythm of each annonaceous species is fundamental to the correct management and conservation of both plant and pollinator beetle species. Finally, this study provides information relevant to extractive economic of *A*. *coriacea*.
